# A Third Measure-Metastable State in the Dynamics of Spontaneous Shape Change in Healthy Human's White Cells

**DOI:** 10.1371/journal.pcbi.1001117

**Published:** 2011-04-07

**Authors:** Karen A. Selz

**Affiliations:** 1 Fetzer Franklin Laboratory of the Cielo Institute, Asheville, North Carolina, United States of America; 2 Emory University School of Medicine, Atlanta, Georgia, United States of America; Physical Sciences Division, Office of Naval Research, United States of America

## Abstract

Human polymorphonuclear leucocytes, PMN, are highly motile cells with average 12-15 µm diameters and prominent, loboid nuclei. They are produced in the bone marrow, are essential for host defense, and are the most populous of white blood cell types. PMN also participate in acute and chronic inflammatory processes, in the regulation of the immune response, in angiogenesis, and interact with tumors. To accommodate these varied functions, their behavior is adaptive, but still definable in terms of a set of behavioral states. PMN morphodynamics have generally involved a non-equilibrium stationary, spheroid *Idling* state that transitions to an activated, ellipsoid translocating state in response to chemical signals. These two behavioral shape-states, spheroid and ellipsoid, are generally recognized as making up the vocabulary of a healthy PMN. A third, “random” state has occasionally been reported as associated with disease states. I have observed this third, *Treadmilling* state, in PMN from healthy subjects, the cells demonstrating metastable dynamical behaviors known to anticipate phase transitions in mathematical, physical, and biological systems. For this study, human PMN were microscopically imaged and analyzed as single living cells. I used a microscope with a novel high aperture, cardioid annular condenser with better than 100 nanometer resolution of simultaneous, mixed dark field and intrinsic fluorescent images to record shape changes in 189 living PMNs. Relative radial roundness, *R*(t), served as a computable order parameter. Comparison of *R*(t) series of 10 cells in the *Idling* and 10 in the *Treadmilling* state reveals the robustness of the “random” appearing *Treadmilling* state, and the emergence of behaviors observed in the neighborhood of global state transitions, including increased correlation length and variance (divergence), sudden jumps, mixed phases, bimodality, power spectral scaling and temporal slowing. Wavelet transformation of an *R*(t) series of an *Idling* to *Treadmilling* state change, demonstrated behaviors concomitant with the observed transition.

## Author Summary

Human white blood cells, polymorphonuclear leucocytes (PMN), were microscopically imaged and analyzed as single living cells. PMN are generally observed in a spheroid *Idling* state transitioning to an activated, egg-shaped, translocating state when triggered by the body's signals of infection or inflammation. Occasionally, PMN are observed in a third behavioral state that looks like dancing in place, with protrusions thrown out and retracted, sometimes several simultaneously, in apparently random directions. This behavior previously had been thought to be associated with disease. Here this third state, that I call *Treadmilling*, is a relatively common way that PMN from healthy people get “stuck” in an intermediate phase. Relative radial roundness, *R*(t), served as a computable order parameter, and time series of *R*(t) were derived from microscopic image series of each of 189 PMN. Only *R*(t) series from cells that stayed healthy, maintained a single behavioral state and did not have contact with other bodies for the 30 min recording period were analyzed further. Comparison of measures made on the *R*(t) series of cells in the *Idling* versus *Treadmilling* states quantitatively distinguish states and suggest behavior in the vicinity of global state transitions. Wavelet transformation of an *R*(t) series of a captured state change supports this finding.

## Introduction

Polymorphonuclear neutrophil granulocytes (PMNs) are the body's most abundant class of white blood cells. At circulating cell levels of ∼10^11^, PMNs make up about 62% of all human white cells [1]. An idling, non-activated neutrophil circulates in the blood for 8 to 12 hours before undergoing apoptosis. When stimulated by inflammatory cytokine signals released by endothelial, mast cells and/or macrophages, PMNs can survive for 24 to 48 hours [2]–[4].

Once activated, idling PMNs exploit shape change dynamics as they tether to the endothelium of post-capillary venules, making and breaking bonds while rolling along the venule's endothelial bed of selectins. Following concentration gradients of inflammatory ligands, they wriggle through the vessel wall and migrate to the site of the initiating inflammation. The motions accompanying this migration exploit the PMN's cytoskeletal, actin polymerization-depolymerization cycle that configures the dynamics of shape change and translation [5], [6].

The behavioral dynamics of two of the shape changing metastable states and their associated translational motions are well established. PMNs manifest these metastable shape-motional states *in vivo* and *in vitro*. They are: (1) The circular-spherical, *Idling* state manifesting standing waves and fast and fine random fluctuations in the leucocyte's apron edge; (2) The activated, ellipsoid, polarized migrating state, with almost exclusively positive gradient-directed lamelopodia and filopodia formation [7], [8].

The complexity of the scenario described above and the necessity of as many as 100 distinct protein/protein interactions to coordinate the actin cytoskeletal apparatus alone, prompts both a phenomenological approach and the consideration of a potentially larger set of shape-motional states which may be transitional, non-stationary and not easily quantified.

In addition to the metastable spherical-round, *Idling* and elliptical-migratory state, I have found a statistically prominent, third, measure metastable state in the PMNs from the fresh blood of healthy human subjects. This non-translating, high amplitude, shape changing state has been previously observed and interpreted as a functionally disordered manifestation of immunological and hematological pathology [9]–[14]. The goals of these studies are to identify, quantitatively characterize and discriminate this third shape-motional PMN state from the other two. I call this third dynamical state, *Treadmilling*, the word used to also describe a key part of the underlying F,G-actin dynamics [15]. I have observed the *Treadmilling* state in 12 of the 18 healthy, adult volunteers contributing blood for this study.

### A Statistical Concern

We remind ourselves that there are neurobiological limits on the both the resolution afforded by empirically meaningful partitions [16], and the minimal length of a neurobiologically defensible time series [17]. In these studies I must be concerned with how long the cells being observed maintain their anatomical and functional integrity and within that viability limit, how many observations I can make without the vacuous artifice of over-sampling.

The issue more generally is the selection of an appropriate sample size of an intrinsically non-stationary system. Counter-intuitively, it has been shown that under certain conditions of limited information, repeated too-short sample lengths come to be computationally superior globally [18].

In the past I have dealt with this problem by studying repeated time series derived measures yielding populations of not necessarily convergent estimates [19] with, nonetheless, distributional properties of the measures, such that I can estimate each measure's central and higher moments, range of variation and statistical differences between measures in comparisons of varying observed system state [20]. This is the approach to be taken here.

## Methods

### Relative Radial Roundness, *R*(t), as an Order Parameter

It is difficult to find a global quantitative measure on the dynamics of emergent phenomenon with the nice properties of additivity, continuity and differentiability [21], [22]. Such a measure has been called an *order parameter*, named for its use in tracking a system's dynamics through transitions in the system's degree of order. In gas-liquid transitions, the order parameter is density [23], while in ferromagnet transitions, for instance, it is net magnetization [23], [24]. Perhaps the best known example of an order parameter is relative phase, the Landau-Ginsberg order parameter [23], [24], used in the phenomenological description of thermodynamic and superconducting transitions [25].

I have examined the autonomous, time-dependent shape changes of individual *Idling* and *Treadmilling* human white cells as real, spatially extended dynamical systems. I use a global measure on the PMN's *relative radial roundness*, *R*(t), as the order parameter. *R*(t) = *r*_1_/*r*_2_ is computed as the ratio of the radius of the cell assumed to be an ideal circle, *r_1_ = p/2π* in which *p* is the sum of the pixels outlining the cell's *perimeter* and *r*_2_ = (A/π)^0.5^ using the sum of the pixels within the cell's silhouette as the cell's *area*, *A. R*(t) = *r*_1_(t)/*r*_2_(t) = {*p*/2π/(A/π)^0.50^}(t) computed at each time step. If both *r*_1_ and *r*_2_ were derived from an abstract, idealized circle, *R*(t) = *r*_1_/*r*_2_ = 1.0, such that *log*{*R*(t) = *r*_1_/*r*_2_} = 0, the characteristic lower limit of a generic order parameter. Deviations from this reference characterize changes in state [21]–[23], [25]. My use of the global order parameter, *R*(t), contrasts with a previous use of an averaged local measure, the power spectral transformation of a sequence of angles resulting from the piece wise linear segmentation of the cell's circumference [26]. The use of *R*(t) more closely resembles a differential geometric pattern map [27].

### Experimental Procedures

One hundred and eighty-nine PMNs from fifty-three peripheral blood samples were collected from 18 healthy adult volunteers, aged 26 to 72. The blood samples were allowed to sediment gravitationally for 40 minutes at room temperature. A population of PMNs (and other white cells and platelets) were removed from the buffy coat by micropipette and, along with associated plasma, placed within a 12 mm ring painted on a glass slide, forming a ∼20–25 µm deep well, compared to the average 5.7 µm vertical space between a plain slide and its cover slip [28]. The 5.7 µm gap of standard slides and coverslips is considerably smaller than the average diameter of PMNs, leading to some mechanical compression of the cell contributing to their activation, and allowing the cell to move along the slide substrate and cover slip simultaneously [28]. The slides used in this study do not suffer from these deficits.

PMN autonomous motions were observed using an Olympus BX41 microscope fitted with CytoViva dark field and fluorescent optical illumination systems, which includes a unique, high-aperture, cardioid annular condenser (www.scitech.com.au). The CytoViva condenser makes it possible to visualize objects of below 100 nm in diameter in real time, and with the cellular samples in an unfixed, living, active state [29], [30]. Because PMNs were treated gently, avoiding perturbations of column separation and elution, it became possible to reliably study a PMN continuously for 30 to 60 minutes before the onset of granular clumping, membrane blebbing and other signs of nascent apoptosis [31]. Data collection continued until ten each *Idling* and *Treadmilling* cells met the conditions for inclusion in the study. Specifically, only cells that maintained healthy, one state behavior, and did not have contact with any extracellular objects for the entire 30 min recording period were retained for analysis.

**Figure 1:**
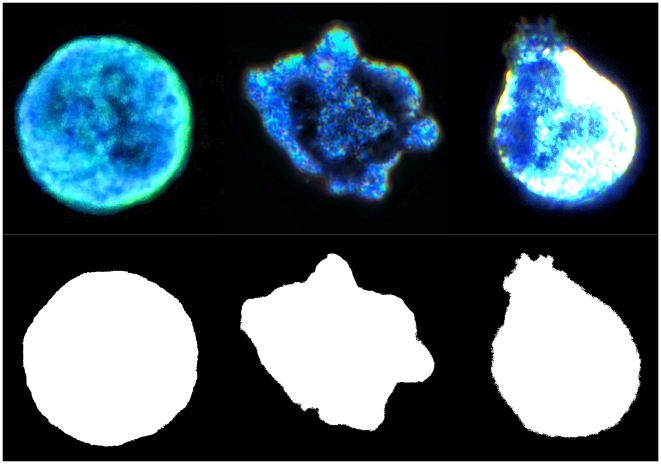
Representative binary color coded silhouettes of PMNs in round *Idling*, *Treadmilling* and elliptically polarized/translocating shape-states.

*Idling* PMNs are characterized by their near spheroid shape (quasi-circular in two dimensions). In this state, the microscopically visible autonomous motions are limited to standing waves on the cell surface and low amplitude fluctuations of the cell's microvillus border. In contrast, *Treadmilling* PMNs demonstrate large and irregular changes in cell shape. Multiple transient, often simultaneously appearing, pseudopodia and lamelopodia emerge from the cell surface, oriented apparently randomly and without significant movement of the PMNs center of mass. The cell's movement was less than 1.5 times the maximum diameter of the cell over the typical ∼30 minute recording sessions. **Figure 1** portrays binary color coded, characteristic silhouettes of the round *Idling*, *Treadmilling* and elliptically polarized/translocating shape-states of PMNs. Only videos micrographs of mature, segmented neutrophils that did not have contact with any cells or extracellular entities and remained visibly healthy over the thirty minutes of observation, in addition to manifesting stable state behavior were retained for further analysis.

Images were collected every 2 sec for 30 min using an Optronics Microfire 1200×1600 CCD array camera [30] resulting in a 900 point *R*(t) series of high resolution images per PMN. The slowness of the cell shape changing motions led to the finding that more frequent sampling within the time limit of cellular integrity was obviously redundant. In the geometric computations, each primary image was used to produce two binary, 0,1, digital daughter images: an area map of *A*, and perimeter map of *p*. The 0,1 coding of the pixels of the two daughter images were converted into binary arrays and used compute the *R*(t) time series.

### Computation of the Measures on *R*(t)

In light of the above discussion of biological constraints on sample length and the intrinsic non-stationarity of the PMNs shape motion series, statistical distributions of often individually non-convergent measures made on each of the cells, serve as the basis for comparisons of *Idling* and *Treadmilling* states. Statistical evaluations are then made on populations of possibly incomplete measures, not on the raw observations. Rules of thumb concerning sample length requirements for any particular measure [32] though easily attainable in physical and computational systems, often ignore the intrinsic series limits and non-stationarity of real, behaving, biological systems. In addition to the use of the distribution of each particular kind of measure, I study an aggregate of several, often incomplete measures, each reflecting different aspects of the shape-motional dynamics of PMNs.

On the *R*(t) of each cell, I study: (1) The central moments of the *R*(t) distribution, the mean *S*_1_ and standard deviation, *S*_2_, as well as the skewness, *S*_3_, indicating the asymmetry of the density distribution of *R*(t), estimated using the third moment, *m_3_*, divided by the cubed root of the variance squared, *S*_3_ = *m_3_/var^3/2^*. The kurtosis, *S*_4_, of *R*(t) is computed using the relation, S_4_ = m_4_/variance^2^ -3 [33]; (2) An estimate of the *R*(t)'s orbital divergence, its sensitivity to initial conditions, in a three dimensional embedding space, was computed using a generic algorithm for the leading Lyapounov exponent, Λ_1_ [34]; (3) Differences in a hierarchical scaling property of *R*(t), by computing the scaling exponent α derived from its power (frequency)spectrum, as the slope of the middle third of the linear best fit of the log power-log frequency relation [35]; (4) An example of the time dependence of the scaling of *R*(t) was estimated from a Morlet continuous wavelet transformation using standard algorithms [36]–[38].

### Visualizing Phase Space Behavior of *R*(t)

To visualize the phase space behavior I used relatively denoised, three dimensional Broomhead-King, B/K, eigenfunction, Ψ_i_ embedding of the *R*(t)s. To do this, I computed and plotted Ψ_1_, Ψ_2_, and Ψ_3_ with respect to each other [39], [40]. Each *R*(t) series generated an M-lagged data matrix on which an MxM Hermitean autocovariance, C_M_, matrix was computed, with M = 8, a typical correlation decay interval. C_M_ was then decomposed into its eigenvalue-ordered eigenvectors. The eigenvectors associated with the three largest eigenvalues were each composed with the original *R*(t) series to form B/K eigenfunctions Ψ_1_, Ψ_2_, and Ψ_3_. These formed the axes of the B/K eigenspace reconstruction. Because *R*(t) behavior attributable to the lower, excluded, eigenvectors accounts for the trivial, “noise” component of the variance, the resulting *eigenfunction space embedding* (each successive point being a triple) is relatively denoised compared with the more commonly used phase delay space construction [41].

Another graphical representation of the orbital behavior of *R*(t) is its two dimensional, *i* = τ_1_, *j* = τ_2_, Recurrence Plot, RP[*R*(t)]_i,j_, introduced by Eckmann [42]. Graphical representations of RP[*R*(t)]_i,j_ are two dimensional lattices, each point computed as RP_i,j_ = Θ(ε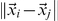), *_i,j = 1…N_*, where 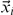 in R^2^ represents the location of the orbit in phase space at time *i*. 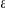 is the static distance defining the “closeness” threshold, and Θ is the Heavyside function. The resulting binary series, each point ε- close or not to the previous value, is coded in black and white. Here, a standard time delay three dimensional embedding was used, with delay τ = 1 [43]. If 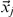 falls within the distance 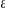 of 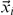, 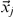 is considered to be a recurrence of 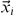, otherwise not. Clustering in RP_i,j_ has been used to discriminate among three characteristic patterns of intermittency [44].

## Results

There were highly significant differences between the measures *S_2_*, *S_3_*, Λ_1_, and α that were made on the *R*(t) series of the PMNs in the *Idling* versus the *Treadmilling* state, see **Table 1**. No significant differences were found between the two distributions of *S*_1_ or *S*_4_.

**Table 1:** Measure averages for cells in each state group.

Idling (n = 10)	Treadmilling (n = 10)	t(df); ρ
Mean = 2.935	Mean = 2.932	t_(18)_ = 0.004; ρ<0.4983
SD = 0.1023	SD = 0.3882	t_(18)_ = 4.816; ρ<0.0001
Skew = 0.2993	Skew = 0.7273	t_(18)_ = 2.419; ρ<0.0132
Kurtosis = 0.7229	Kurtosis = 1.165	t_(18)_ = 0.652; ρ = 0.2612
λ_1_ = 0.6242	λ_1_ = 0.5066	t_(18)_ = 2.592; ρ<0.0090
α = −0.4561	α = −1.0290	t_(18)_ = 10.600; ρ<0.0001

**Table 1** reports the results of measures made on the *R*(t) series of ten *Idling* PMN and ten *Treadmilling* PMN, all of which had remained healthy and in a single behavioral state for the 30 min recording period and made no contact with extracellular bodies during that time.

The qualitative differences in the shape-motional patterns implied by the statistically significant differences in the measures in **Table 1** are consistent with behavior that was observed microscopically in the two pre-polarized states: (1) The small, stochastically wavy border fluctuations in cell shape of the generic *Idling* PMNs; (2) A range of large, simultaneously multiscale motions in cell shape variations of *R*(t) in the *Treadmilling* state. For examples, compared with *Idling*, the increase in asymmetric amplitude in *Treadmilling* is reflected in increases in *S*_2_ *and S*_3_, and the increase in shape-motional order in *Treadmilling* is seen in the statistically significant decrease in Λ_1_, the leading Lyapounov index of expansive, orbital mixing [45]. The larger, smoother, more correlated shape motions of the *Treadmilling* state are seen in statistically significant increases in α in the *Treadmilling* versus *Idling* states. Without a significant difference in the means of *R*(t), the variational measures make the discrimination between *Idling* and *Treadmilling* states.

**Figure 2:**
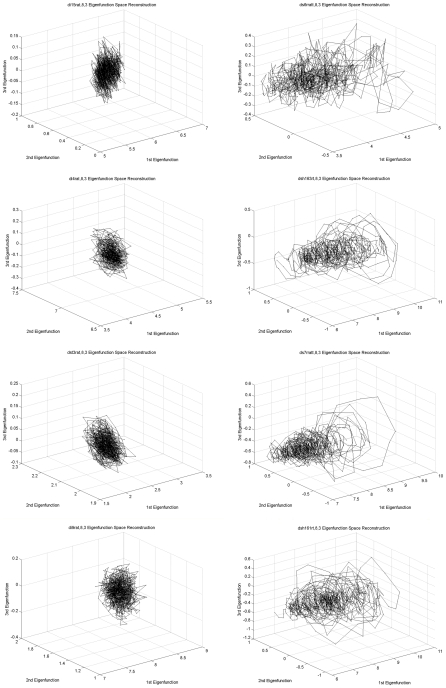
Shows the *eigenfunction space embedding* (see text) for four representative *Idling* cells (left column) and four *Treadmilling* cells (right column).

Consistent with the differences in behavior described by direct observation and the aggregate of measures (see **Table 1**), **Figure 2** portrays the previously described {Ψ_1_, Ψ_2_, Ψ_3_}_1…900_ B/K eigenfunctions embedding of four representative *Idling* cells (left column) and four *Treadmilling* cells (right column). The phase portraits of the *Idling* cells reflect symmetric, small, random fluctuations around a near stationary state. *Treadmilling* cells manifest larger, more irregular, asymmetric phase space motions which occupy almost an order of magnitude larger volume than that by the *Idling* state.

**Figure 3:**
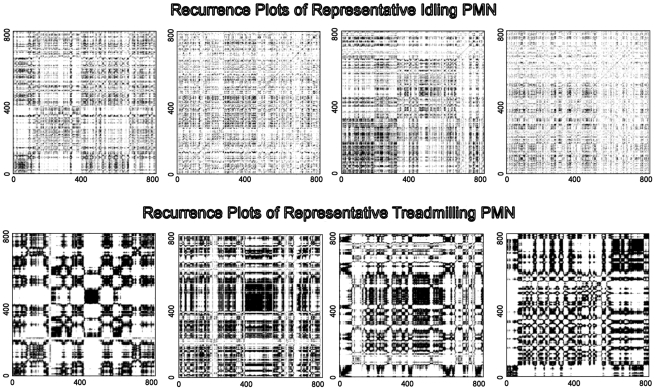
Contains recurrence plots, RP_i,j_, of four representative PMNs in the *Idling* state (top row) and four in the *Treadmilling* state (bottom row). ε = 1 for all plots.

Another geometric, graphical treatment of the cell's shape motional behavior is displayed in **Figure 3**. We see the recurrence plots, RP[*R*(t)]_i,j_ of the four representative PMNs in the *Idling* state (top row) and four in the *Treadmilling* state (bottom row), in which ε = 1 for all plots. The RP[*R*(t)]_i,j_ of the *Idling* cells demonstrate more homogeneous temporal distributions of returns typical of more random data with shorter correlation lengths/relaxation times. The square patches of only lightly increased density overlaid on the more uniform surround are consistent with both the visualized small amplitude oscillations in *R*(t) in the *Idling* state and with the statistical results reported in **Table 1**. The RP[*R*(t)]_i,j_ of cells in the *Treadmilling* state are, as expected, less homogenously distributed, manifesting clustering in the return times across multiple times scales, as well as apparent discontinuous changes in their phase space patterns. For example, short interval “bursting” interleaved with low amplitude, long interval behavior is seen in the *Treadmilling* cells' RP[*R*(t)]_i,j_. *Treadmilling* PMNs RP[*R*(t)]_i,j_ portraits are consistent with recurrence patterns of intermittency [44], [46].

**Figure 4:**
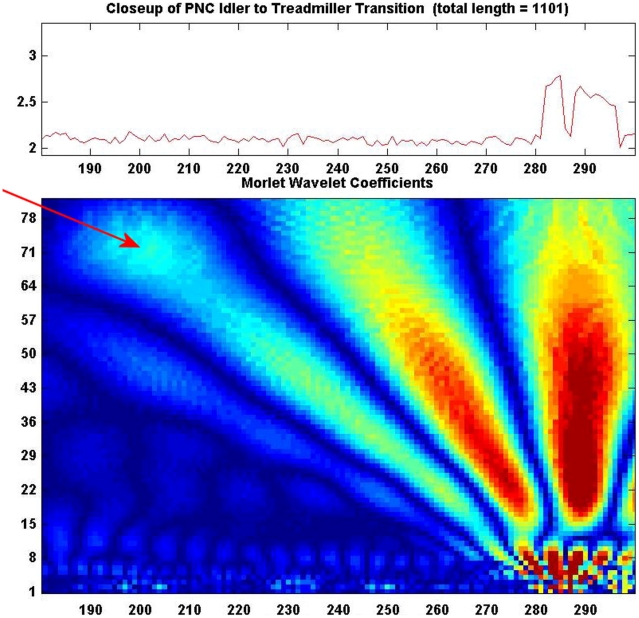
Shows a “close-up” of a PMN during a phase transition. The upper panel shows the *R*(t) series, while the lower panel depicts the continuous 1D Morlet wavelet moduli for that time interval (x-axis). Scale of the basis function increases up the y-axis. The colormap passes through the visible spectrum; blues representing low Morlet moduli amplitudes to high valued reds. The cell is initially *Idling*, and begins *Treadmilling* at data point t = 283. Note that anticipatory slow high amplitude fluctuations begin to appear at about point t = 198.

Four of the seven order parameter measures demonstrated statistically significant differences between the *Idling* and *Treadmilling* PMNs, **Table 1**. While observing and recording the real-time behavior of 189 PMNs, I witnessed many cells transitioning from one state to another among my three defined behavioral regimes. Data series including such transitions were plagued by the same complications as were the single state series (e.g., cell-cell interactions, apoptotic behavior) in addition to too short times in one or more behavioral state to allow any analysis. I was finally able to make sufficient observations portraying a single PMN shape motion transformation in real time. **Figure 4** is a Morlet wavelet graph, in continuous time along the x-axis, and scale (∼wavelength) along the Y axis. **Figure 4** contributes evidence for a continuous transition in shape motion state, here from *Idling* to *Treadmilling*. **Table 2** lists measures before and after this single cell transition. Note that the direction and approximate magnitude of change resemble those of the population of statistically significant values in **Table 1**.

**Table 2:** Measures made on a nutrophil before and after idling to treadmilling state change.

Idling	Treadmilling
Mean = 2.0969	Mean = 2.1614
SD = 0.0348	SD = 0.1620
Skew = −0.1942	Skew = 2.0076
Kurtosis = 2.8552	Kurtosis = 8.0831
λ_1_ = 0.6520	λ_1_ = 0.498
α = −0.5681	α = −1.0486

**Table 2** reports the results of the same measures listed in **Table 1**, this time made on the *R*(t) series of a single PMN first in an *Idling* state and then in a *Treadmilling* state. This PMN also remained healthy for the 30 min recording period and made no contact with extracellular bodies during that time.

## Discussion

There are established physiological mechanisms and behavior that are consistent with both our qualitative microscopic observations and quantitative aggregate measure descriptions. PMNs are known to oscillate on multiple time and space scales, from 7 sec, 70 sec, and 260 sec membrane potential fluctuations [47] and 25 sec calcium flux oscillations [48], to the ∼8 sec bound/unbound actin oscillations [49], to 21.6 sec and 230 sec glycolytic cycles producing NAD(P)H oscillations [47], and 10 sec and 20 sec pericellular proteolysis fluctuations [48], among many others. The *R*(t) series in this study evidenced scaling, board band power spectra with multiple resonances [50]. It is likely some reported modes contribute to the cell shape fluctuations directly and others contribute to the emergence of other dynamical patterns.

The slowest Fourier mode in *S_ω_* [*R*(t)] of the *Idling* state had an average 8.457 minutes oscillation, whereas that of the slowest *S_ω_* [*R*(t)] of the *Treadmilling* state averaged a 4.201 minutes oscillation. It is interesting that these characteristic times correspond roughly to the results of studies of the characteristic remodeling times composed of actin filament diffusion, polymerization and then turnover coordinated with cellular migratory motions [51], [52]. It appears that the transition from *Idling* into the intermittent *Treadmilling* regime occurs as the *Idling* state loses some of its dynamical structural stability, and its shape motion scenario becomes driven by several quasiperiodic, multi-periodic metabolic and physiological cellular oscillator mechanisms [53], [54].

As listed in **Table 1** and **Table 2**, a comparison of *Idling* with *Treadmilling* PMNs reveals significantly different *R*(t) order parameter dynamics. Projected to a two dimensional plane (**Figure 1**), one sees an associated difference in the underlying planar geometry, with the *Idling* PMNs manifesting one centroids in their circularity, and the *Treadmilling* PMNs with two point defined, barycentric ellipses.

Many characteristics of the changes in measures in the distinct single state observations and in the computable, real-time transition from *Idling to Treadmilling* suggest the typical signs of a phase transition [21]–[23], [25]. These included: (1) Increasing amplitude of *R*(t) variability seen in the *S*_2_ and *S*_3_ of the cell shape fluctuations; (2) Decreased leading Λ_1_ becoming less positive in the direction of zero, shadowing the leading eigenvalue of the unknown underlying partial differential equation; (3) An increase in the log-log power spectral scaling index, α, reflecting a “less white” spectral pattern of *R*(t) fluctuations, also consistent with slowing; (4) The Morlet wavelet transformation of a continuous time *R*(t), evidenced anticipatory, high amplitude slowing and a mixed phase regimes in the neighborhood of a real-time PMN shape fluctuation transition. The *eigenfunction space embedding* of the sequence of triples, {Ψ_1_, Ψ_2_, Ψ_3_}_i_ demonstrated directly the space-time morphogenic transformation undergone by *R*(t) in the *Treadmilling* state with reference to that of the *Idling* cell state. Recurrence plots, RP_i,j_ depicted increased phase space clustering consistent with the more hierarchical, intermittent dynamics of the *Treadmilling* PMNs in contrast with the more randomly distributed and metrically transitive space of the *Idling* RP_i,j_. It should be noted that the action spaces of less uniform intermittency and those of more uniform transitivity reflect common metastable alternatives in the dynamics of some biological sciences [48].

Finally, I have spent hundreds of hours microscopically tracking 189 individual PMN cells in the hopes of answering these questions about state and state transitions. While only one such transition was recorded with sufficient observations in both the *Idling* and *Treadmilling* states to allow statistical analyses, many transitions were observed. I have seen *Idling* cells transition to *Treadmilling*, and *Treadmilling* cells ball up and *Idle* (although with slightly ragged aprons). I have also observed numerous instances of *Idling* cells polarizing and *Translocating* until they reach some point at which point they *Idle* again. The only transitions that were not observed were from the *Treadmilling* to the polarized, single lamelopod, *Translocating* state or vice versa. In either case the cells ball-up briefly into an *Idling* appearance before changing again. See **Table 3**.

**Table 3:** Observed cell state transitions.

FromTo	Idling	Treadmilling	Translocating
Idling	**X**	**X**	**X**
Treadmilling	**X**	**X**	
Translocating	**X**		**X**
